# Joint Action: Mental Representations, Shared Information and General Mechanisms for Coordinating with Others

**DOI:** 10.3389/fpsyg.2016.02039

**Published:** 2017-01-04

**Authors:** Cordula Vesper, Ekaterina Abramova, Judith Bütepage, Francesca Ciardo, Benjamin Crossey, Alfred Effenberg, Dayana Hristova, April Karlinsky, Luke McEllin, Sari R. R. Nijssen, Laura Schmitz, Basil Wahn

**Affiliations:** ^1^Department of Cognitive Science, Central European University (CEU)Budapest, Hungary; ^2^Faculty of Philosophy, Theology and Religious Studies and Donders Center for Cognition, Radboud University NijmegenNijmegen, Netherlands; ^3^School of Computer Science and Communication, KTH Royal Institute of TechnologyStockholm, Sweden; ^4^Department of Communication and Economics, University of Modena and Reggio Emilia (UNIMORE)Reggio Emilia, Italy; ^5^School of Psychology, University of BirminghamBirmingham, UK; ^6^Institute of Sports Science, Leibniz University of HannoverHannover, Germany; ^7^Faculty of Psychology, University of ViennaVienna, Austria; ^8^School of Kinesiology, University of British Columbia (UBC)Vancouver, BC, Canada; ^9^Behavioural Science Institute, Radboud University NijmegenNijmegen, Netherlands; ^10^Institute of Cognitive Science, University of OsnabrückOsnabrück, Germany

**Keywords:** joint action, social interaction, action prediction, joint attention, culture, sensorimotor communication, coordination

## Abstract

In joint action, multiple people coordinate their actions to perform a task together. This often requires precise temporal and spatial coordination. How do co-actors achieve this? How do they coordinate their actions toward a shared task goal? Here, we provide an overview of the mental representations involved in joint action, discuss how co-actors share sensorimotor information and what general mechanisms support coordination with others. By deliberately extending the review to aspects such as the cultural context in which a joint action takes place, we pay tribute to the complex and variable nature of this social phenomenon.

## Acting in a Social World

**FIGURE 1:**
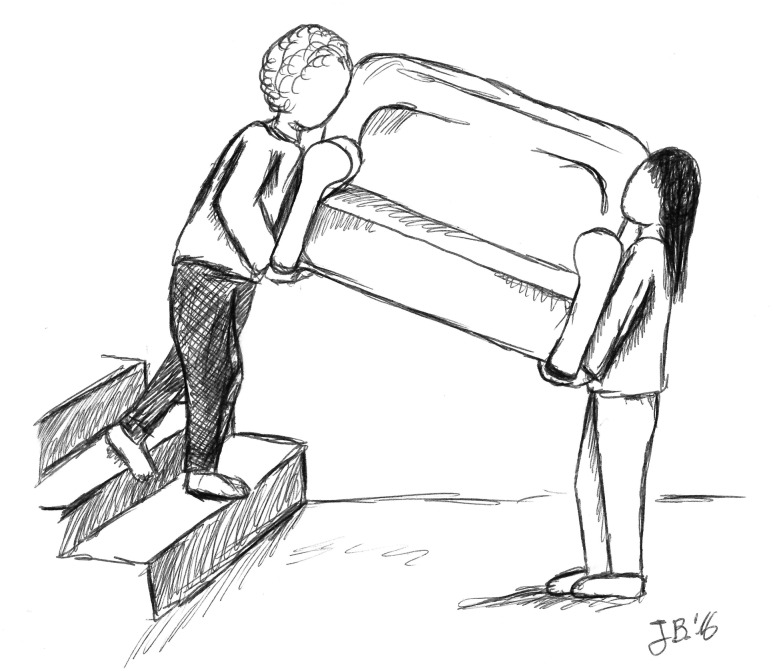
**Two people carrying a heavy sofa together face the challenge of coordinating their actions in a temporally and spatially precise manner**.

People rarely act in isolation; instead, they constantly interact with and coordinate their actions with the people around them. Examples of such ‘joint actions’ range from carrying a sofa with multiple people (**Figure 1**), building a toy brick tower with a child, playing basketball, to performing a musical duet. Accordingly, an often-used definition describes joint action as “any form of social interaction whereby two or more individuals coordinate their actions in space and time to bring about a change in the environment” (Sebanz et al., 2006, p. 70). Especially in light of a long research tradition that focused on the psychological (neuro)cognitive and perceptual processes of individuals, it is crucial to realize the importance of studying these processes in the social environment in which they typically occur.

## Coordination Mechanisms

Agents involved in joint action make use of different mechanisms to support their coordination. These include forming representations about, and keeping track of, a joint action goal and the specific to-be-performed tasks. Some coordination mechanisms depend on sensorimotor information shared between co-actors, thereby making joint attention, prediction, non-verbal communication, or the sharing of emotional states possible. General mechanisms, which depend to a lesser degree on sharing information online, influence and support coordination such as when co-actors rely on ‘coordination smoothers’ or conventions to act together. This article provides an overview of these coordination mechanisms (**Table 1**) and their role for joint action. We focus on intentional real-time joint action, in which adult co-actors share a physical space and where coordination may require high temporal precision. It therefore complements work on rhythmic or unintentional coordination (Repp and Keller, 2004; Schmidt and Richardson, 2008), verbal communication (Clark, 1996; Brennan et al., 2010), strategic cooperation (Schelling, 1960), and coordination in more temporally or spatially remote joint tasks.

**Table 1:** Overview of different coordination mechanisms supporting joint action, along with a set of examples.

Coordination mechanism	Example
**Mental representations in joint action**	
Joint action goal	Relocating a sofa by lifting and moving it together
Task (co-)representation	Carrying a sofa forward or backward
Monitoring	Noticing errors in a co-actor’s performance
**Sharing sensorimotor information**	
Joint attention and shared gaze	Being mutually aware of an obstacle in the way
Sensorimotor prediction	Predicting a co-actor’s movement direction
Sensorimotor communication	Pushing a co-actor into a certain direction
Haptic coupling	Feeling a co-actor pushing the sofa
Multisensory processing	Integrating information from different senses
Emotion understanding and expression	Realizing how exhausted a co-actor is
**General mechanisms supporting coordination**	
Coordination smoothers	Distributing the task of moving forward or backward
Affordances	Being constrained by available space and a co-actor’s physical strength
Conventions and culture	Appreciating rules about who carries more weight

## Mental Representations in Joint Action

The following subsections discuss the mental representations underlying joint action, such as representing and monitoring the joint action goal and agents’ specific tasks.

### Joint Action Goal

To successfully perform a joint action, actors need to plan their own action in relation to the desired outcome and/or their co-actor’s actions. For example, Kourtis et al. (2014) showed that the neural signature of action planning is modulated when one’s own action is part of a joint plan, e.g., when clinking glasses with another person, compared to performing a corresponding solo or bimanual action. According to a minimal joint action account, agents intending to perform a coordinated action with others minimally represent the joint action goal and the fact that they will achieve this goal with others (Vesper et al., 2010). This does not presuppose high-level interlocking mental representations (cf. Bratman, 1992), and might therefore form the basis for joint action in young children (Butterfill, 2012). Moreover, joint action goals influence the acquisition of new skills: after learning to play melodies in a joint action context (i.e., duets), piano novices played better when later coordinating toward the shared action goal (the duet) compared to their own action goal (the melody; Loehr and Vesper, 2016). Further evidence for the role of goal representations in joint action comes from work on complementary action (Sartori and Betti, 2015) showing that, in contrast to an imitation context, performance of an action is facilitated if the goal is to complement someone else (van Schie et al., 2008; Poljac et al., 2009).

### Task (Co-)representation

In many joint actions, detailed knowledge about another’s task is available and people tend to co-represent these tasks, even if detrimental to their own action performance. For instance, one might be influenced by others’ stimulus-response rules in reaction-time tasks (Sebanz et al., 2003, 2005) or automatically memorize word list items relevant only to another person (Eskenazi et al., 2014). When acting together with others toward a joint goal, representing a co-actor’s task can be beneficial as it enables agents to predict others’ actions and to integrate them into their own action plan. For example, knowledge about a co-actor’s task can be useful if access to online perceptual information about the co-actor’s action is unavailable such that monitoring the co-actor’s unfolding action and continuously adjusting in an appropriate manner is not possible. This was shown in a study where dyads coordinated forward jumps of different distances such that they would land at the same time (Vesper et al., 2013). Although co-actors could not see or hear each other’s actions, task knowledge about the distance of each other’s jump was sufficient to predict the partner’s timing and adjust their own jumping accordingly.

### Monitoring

While performing a joint task, co-actors typically monitor their task progress to determine whether the current state of the joint action and the desired action outcome are aligned (Vesper et al., 2010; Keller, 2012). For example, one might keep track of how far a jointly carried sofa has been moved and whether all task partners are equally contributing to lifting the weight. Monitoring is useful to detect mistakes or unexpected outcomes in one’s own or one’s partner’s performance, enabling one to quickly react and adapt accordingly. Performance monitoring in social contexts involves specific processes and brain structures such as brain areas involved in mentalizing and perspective-taking, e.g., medial prefrontal cortex (van Schie et al., 2004; Newman-Norlund et al., 2009; Radke et al., 2011). Findings from an EEG experiment with expert musicians (Loehr et al., 2013) indicate that the neural signature associated with the detection of unexpected musical outcomes is similar irrespective of whether an auditory deviation arises from one’s own or the partner’s action. This suggests that co-actors monitor the actions toward the overall joint goal in addition to their own individually controlled part.

## Sharing Sensorimotor Information

The following subsections provide an overview of different ways in which co-actors share sensorimotor information to support joint action through joint attention, prediction, non-verbal communication, or sharing emotions.

### Joint Attention and Shared Gaze

Others’ eye movements are an important source of information about what others see and about their internal states (Tomasello et al., 2005). For example, when jointly moving a sofa, co-actors may use mutual gaze to infer whether everyone is aware of a potential obstacle that is in their way (e.g., a curious dog). Joint attention relies on co-actors’ ability to monitor each other’s gaze and attentional states (Emery, 2000). For instance, when synchronizing actions, co-actors divide attention between locations relevant for their own and for their co-actor’s goal (Kourtis et al., 2014; see Böckler et al., 2012; Ciardo et al., 2016 for similar results using different tasks), and sharing gaze affects object processing by making attended objects motorically and emotionally more relevant (Becchio et al., 2008; Innocenti et al., 2012; Scorolli et al., 2014). Moreover, in a joint search task, co-actors who mutually received information about each other’s gaze location via different sensory modalities (i.e., vision, audition, and touch) searched faster than without such information (Brennan et al., 2008; Wahn et al., 2015). Together, these findings demonstrate the important role of gaze information for joint action.

### Sensorimotor Prediction

Predicting others’ actions and their perceptual consequences is often important for joint action. When moving a sofa together with someone, individuals need to predict what the other is going to do next in order to adapt their own action and thereby facilitate coordination. It has been postulated that action prediction relies on individuals’ own motor plans and goals such that when an interaction partner’s actions are observed, this activates representations of corresponding perceptual and motor programs in the perceiver (Prinz, 1997; Blakemore and Decety, 2001; Wolpert et al., 2003; Wilson and Knoblich, 2005; Catmur et al., 2007). At a functional level, action prediction can be explained in terms of internal forward models that generate expectations about the sensory consequences of partner-generated actions based on an individual’s own motor experience. At a neurophysiological level, the mirror system (Rizzolatti and Sinigaglia, 2010) provides a plausible mechanism linking action observation, imagination, and representation of others’ actions with motor performance. Although motor prediction has mostly been studied in action observation, some evidence demonstrates that it supports joint action by allowing precise temporal coordination (Vesper et al., 2013, 2014) and that it is modulated by own action experience. For instance, Tomeo et al. (2012) found that expert soccer players, compared to novices, more effectively predict the direction of a kick from another person’s body kinematics (see Aglioti et al., 2008; Mulligan et al., 2016, for similar results with basketball and dart players). Action prediction also affects perception (Springer et al., 2011) as predictions based on knowing another person’s task can bias how their subsequent actions are perceived (Hudson et al., 2016a,b). Due to the overlap of own and others’ sensorimotor representations, additional processes are needed to keep a distinction between self and other (Novembre et al., 2012; Sowden and Catmur, 2015) and to inhibit the tendency to automatically imitate another’s (incongruent) action (Ubaldi et al., 2015).

### Sensorimotor Communication

In some joint actions, it is useful to not only gather information about other people but to actively provide others with information about one’s own actions. Accordingly, co-actors might adjust the kinematic features of their action (e.g., velocity or movement height) in order to make their own actions easier to predict for another person. Thus, ‘sensorimotor communication’ is characterized by having both an instrumental (e.g., pushing a sofa) and a communicative goal (e.g., informing a partner about one’s movement direction). This facilitates action prediction by disambiguating different motor intentions for the observer (Pezzulo et al., 2013), thereby relying on people’s ability to detect even subtle kinematic cues (Sartori et al., 2011). Studies on sensorimotor communication typically involve tasks where a ‘leader’ participant has information about an aspect of a joint task that a ‘follower’ participant lacks and so the follower has to rely on the leader’s action cues to act appropriately. For example, leaders exaggerated the height of their movements to allow followers to more easily recognize the intended action target (Vesper and Richardson, 2014). Similarly, leaders communicate the end-point of a grasping action with the help of exaggerated kinematic parameters, such as wrist height and grip aperture (Sacheli et al., 2013).

### Haptic Coupling

Information about another person’s action might also be provided through the tactile channel. For instance, jointly carrying a sofa allows mutual exchange of force information, revealing co-actors’ movement direction or speed. Accordingly, dyads who performed a joint pole-balancing task enhanced the force feedback between each other to support smooth interaction (van der Wel et al., 2011). Generally, touch can function as an information channel when joint action partners are in physical contact with each other. The ability to decode signals such as emotional cues (Hertenstein et al., 2009) from close physical interaction with their parents is a crucial aspect of children’s development, establishing and regulating social encounters (Feldman et al., 2003). Mother-infant tactile communication, gaze, and emotional vocalization are found in all cultures and societies, although cross-cultural research revealed that touch plays a more important role for communication during play and learning in traditional compared to Western societies (Richter, 1995). Moreover, tactile communication is integral to cultural practices such as dance and martial arts (Kimmel, 2009).

### Multisensory Processing

Information processing in joint action is not limited to only one sensory modality: when carrying a sofa together, visual, auditory, and haptic sensory input is available, facilitating, e.g., the prediction of a partner’s change in movement direction. A recent study provides support for the flexibility of multisensory processing: using a ‘sonification’ technique, in which kinematic movement parameters are transformed into sound, it was shown that ‘sonified’ forces and movement amplitudes on a rowing ergometer provide sufficient information for listeners to predict a virtual boat’s velocity and to reliably discriminate own actions from those of other persons (Schmitz and Effenberg, 2012). Humans are also able to integrate redundant information from multiple sensory modalities, thereby enhancing the reliability and precision of perception (Ernst and Banks, 2002; Wahn and König, 2015, 2016). For instance, whilst the mirror system is mostly understood as a visual system sensitive to biological motion information, it is actually also tuned to auditory (Kohler et al., 2002; Bidet-Caulet et al., 2005) and audiovisual information (Lahav et al., 2007). Neuroimaging evidence shows enhanced activation of most parts of the action observation system (medial and superior temporal sulcus, inferior parietal cortex, premotor regions, and subcortical structures) when observing agents’ convergent compared to divergent audiovisual movement patterns (Schmitz et al., 2013).

### Emotion Understanding and Expression

Sharing emotions with others provides motivational cues helpful to initiate and continue joint tasks and to facilitate coordination (Michael, 2011). Humans are capable of reading others’ affective states from body movements, body posture, gestures, facial expressions, and action performance, possibly via activation of the observer’s corresponding states (Bastiaansen et al., 2009; Borgomaneri et al., 2012). A two-system model of emotional body language (de Gelder, 2006) distinguishes between automatic, reflexes-based manifestations of an emotional message and more deliberate emotional expression based on reflection and decision-making. Together, these efficiently provide information about others’ emotional states and help establish and maintain joint action. For example, having an uncooperative co-actor affected participants’ own response times (Hommel et al., 2009), suggesting that people adjust their own behavior according to the perceived affective states of others. Emotional body language also plays a major role in art improvisations, such as contact improvisation dance (Smith, 2014). Since improvisers explicitly use input from their partners to develop their movement interaction, this dance form allows performers to display and experiment with inner states and emotional body language, which, in turn, influences the overall joint action outcome.

## General Mechanisms Supporting Coordination

The following subsections introduce coordination mechanisms that depend to a lesser degree on shared online information but influence and support joint action more generally.

### Coordination Smoothers

When shared perceptual information is scarce or unavailable, ‘coordination smoothers’ (Vesper et al., 2010) support joint actions. One example is reducing the temporal variability of one’s own actions, first identified in dyads who synchronized the timing of key presses in a reaction time task (Vesper et al., 2011). Co-actors’ responses were overall faster and less variable in joint compared to individual performance and variability reduction effectively improved coordination. A further coordination smoother is the distribution of tasks between joint action partners. In order to facilitate coordination, co-actors who have a relatively easier task might adapt their actions in a different way than those with a more difficult task (Vesper et al., 2013; Skewes et al., 2015). For example, if a door needs to be opened while carrying a sofa to another room, it will be done by the actor who is closer to the door while the other will momentarily take over more weight to provide support.

### Affordances

Affordances are action possibilities available to an agent in an environment (Gibson, 1979). In the context of joint action, information comes from the co-actor’s body or movements and from the objects in the environment in which the joint action takes place. On the one hand, ‘affordances for another person’ specify co-actors’ action possibilities provided by their particular abilities and the environment. For example, based on the perceived relation between chair height and an actor’s leg length, observers can distinguish between maximum and preferred sitting heights of actors of different body height (Stoffregen et al., 1999). Such information is useful in understanding other agents (see Bach et al., 2014, for a review on affordance in action observation) but can also help to efficiently complement their behavior. On the other hand, ‘affordances for joint action’ (or ‘joint affordances’) concern actions available to multiple agents together. For example, when dyads lifted wooden planks alone or together, they transitioned between these two modes based on a relational measure (the ratio of plank length and both persons’ mean arm span; Isenhower et al., 2010). Social affordances might be directly perceived given that the information is publicly available. Therefore, learning to perceive affordances for others might be a natural consequence of learning to perceive affordances for oneself (Mark, 2007). Consequently, own capabilities and experiences play a role in perceiving affordances for others (Ramenzoni et al., 2008), possibly by activating one’s own motor system (Costantini et al., 2011).

### Conventions and Culture

Cultural and societal norms play a major role in regulating behaviors, social encounters, and cooperation in groups by providing conventions that can reliably guide individual behavior. Generally, culture and conventions depend on establishing and maintaining common ground between the members of a group through shared experiences (Clark, 1996). Culture is both a product of large-scale joint actions, such as celebrations or protests, and it profoundly shapes how people approach joint action in small-scale interpersonal encounters. For example, if a person of a higher social rank performs a joint task with their direct subordinate (e.g., an employer carries the sofa together with an employee), coordination might be influenced by the pre-existing power relation, the established culture (e.g., favoring hierarchical or egalitarian communication; Cheon et al., 2011) and the particular situational context (e.g., formal or informal). Joint actions involving people from different cultural backgrounds are an interesting test case for studying cooperation that is not regulated by the framework of a single culture. Different cultures might promote conflicting approaches to communication, decision making, and coordination (Boyd and Richerson, 2009) and consider different amounts of personal space, gaze, or tactile communication appropriate (Gudykunst et al., 1988). For instance, people from East Asia would typically bow for a formal greeting, whereas European people would shake hands. This cultural difference may result in a failure to perform the planned joint action of greeting properly. Strategies to avoid such unsuccessful coordination, e.g., adopting the partner’s cultural technique or establishing a new ‘third-culture’ way, might be used in a variety of joint tasks.

## Conclusion

The aim of this article was to provide an overview of the major cognitive, sensorimotor, affective, and cultural processes supporting joint action. Given the extent of the phenomena (from moving a sofa to playing in a musical ensemble) as well as the variety of coordination mechanisms underlying joint action (as introduced in this review), we postulate that research on joint action needs to acknowledge the complex and variable nature of this social phenomenon. Consequently, future psychological, cognitive, and neuroscientific research might (1) integrate different lines of research in ecologically valid tasks, (2) specify the relative contribution of particular coordination mechanisms and contextual factors, and (3) set the grounds for an overarching framework that explains how co-actors plan and perform joint actions.

## Author Contributions

All authors contributed to the writing. CV developed the article structure and performed final editing of the text.

## Conflict of Interest Statement

The authors declare that the research was conducted in the absence of any commercial or financial relationships that could be construed as a potential conflict of interest.
